# Oxidized LDL and Fructosamine Associated with Severity of Coronary Artery Atherosclerosis in Insulin Resistant Pigs Fed a High Fat/High NaCl Diet

**DOI:** 10.1371/journal.pone.0132302

**Published:** 2015-07-06

**Authors:** Timothy C. Nichols, Elizabeth P. Merricks, Dwight A. Bellinger, Robin A. Raymer, Jing Yu, Diana Lam, Gary G. Koch, Walker H. Busby, David R. Clemmons

**Affiliations:** 1 Department of Pathology and Laboratory Medicine, University of North Carolina at Chapel Hill, Chapel Hill, North Carolina, United States of America; 2 Department of Medicine, University of North Carolina at Chapel Hill, Chapel Hill, North Carolina, United States of America; 3 Department of Biostatistics, University of North Carolina at Chapel Hill, Chapel Hill, North Carolina, United States of America; Northeast Ohio Medical University, UNITED STATES

## Abstract

**Background:**

Insulin-resistant subjects develop more severe and diffuse coronary artery atherosclerosis than insulin-sensitive controls but the mechanisms that mediate this atherosclerosis phenotype are unknown.

**Research Objective:**

To determine the metabolic parameters that associate with the severity of coronary atherosclerosis in insulin resistant pigs fed a high fat/high NaCl diet.

**Key Methods:**

The primary endpoint was severity of coronary atherosclerosis in adult pigs (*Sus scrofa*, n = 37) fed a high fat diet that also contained high NaCl (56% above recommended levels) for 1 year.

**Principal Findings:**

Twenty pigs developed severe and diffuse distal coronary artery atherosclerosis (i.e., severe = intimal area as a percent medial area > 200% in at least 2 coronary artery cross sections and diffuse distal = intimal area as a percent medial area ≥ 150% over 3 sections separated by 2 cm in the distal half of the coronary artery). The other 17 pigs had substantially less coronary artery atherosclerosis. All 37 pigs had blood pressure in a range that would be considered hypertensive in humans and developed elevations in total and LDL and HDL cholesterol, weight gain, increased backfat, and increased insulin resistance (Bergman Si) without overt diabetes. Insulin resistance was not associated with atherosclerosis severity. Five additional pigs fed regular pig chow also developed increased insulin resistance but essentially no change in the other variables and little to no detectible coronary atherosclerosis. Most importantly, the 20 high fat/high NaCl diet -fed pigs with severe and diffuse distal coronary artery atherosclerosis had substantially greater increases (p< 0.05) in oxidized LDL (oxLDL) and fructosamine consistent with increased protein glycation.

**Conclusion:**

In pigs fed a high fat/high NaCl diet, glycated proteins are induced in the absence of overt diabetes and this degree of increase is associated with the development of severe and diffuse distal coronary artery atherosclerosis.

## Introduction

The increasing prevalence of insulin resistance and type 2 diabetes is likely to be attended by a substantial increase in cardiovascular disease (CVD).[1–3] Insulin resistance (IR) is defined as a decreased biological response to normal concentrations of serum insulin that over time leads to compensatory hyperinsulinemia.[2] Insulin resistant and diabetic humans often develop multiple and severe coronary atherosclerotic lesions that are diffuse in that they involve long arterial segments especially in the distal portions of the coronary arteries.[4–8] In addition, there is more extensive remodeling and fibrous caps are thicker.[5, 7] The resulting small caliber arteries are less amenable to angioplasty, stent placement, surgical reconstruction or bypass.[9] Even when coronary artery stent placement or bypass surgery is feasible, often disease progression outside of the stented segment of the coronary artery or bypass insertion site limits the duration of benefit in patients with IR and diabetes.[10] Remarkably, even the most aggressive medical treatment regimens do not lower the risk for CVD to the non-diabetic level.[9, 11–15] These findings strongly suggest that factors other than absolute glucose concentrations may be activating pathophysiological mechanisms that augment the development of atherosclerosis. Thus, there is a need for a relevant animal model of insulin resistance and/or type 2 diabetes that also exhibits severe and diffuse coronary and aortic atherosclerosis which are associated with identifiable biochemical abnormalities.

Our objective was to investigate whether the severity of atherosclerosis is associated not only with lipoprotein concentrations, weight, blood pressure, biomarkers of inflammation and IR in an animal model but also changes in parameters that measure protein glycation. Our experimental approach was to study normocholesterolemic pigs fed a high fat diet that also contained increased NaCl. Our choice of pigs was driven by the fact that, like humans, they develop coronary artery and aortic atherosclerosis and insulin resistance. In addition, pigs have been used in many studies to define the mechanisms that mediate increased atherosclerosis in diabetes.[16–18] Our results show that the experimental pigs developed varying degrees of coronary atherosclerosis that were grouped as either severe and diffuse distal *or* moderate. Atherosclerosis severity was not associated with increases in body weight, backfat, insulin or glucose levels, insulin resistance, blood pressure, or biomarkers of inflammation. Two other variables, however, that have been associated with increased oxidative protein glycation, oxLDL and fructosamine, were found to be substantially higher in animals with the severe and diffuse distal coronary atherosclerosis phenotype as compared to animals with moderate disease. Thus, although our data are associational and not mechanistic, this paradigm suggests that the increased protein glycation that occurs with feeding a high fat/high NaCl diet may enhance the severity of atherosclerosis development in insulin resistant, hypercholesterolemic pigs.

## Materials and Methods

### Experimental Pigs

**Fig 1:**
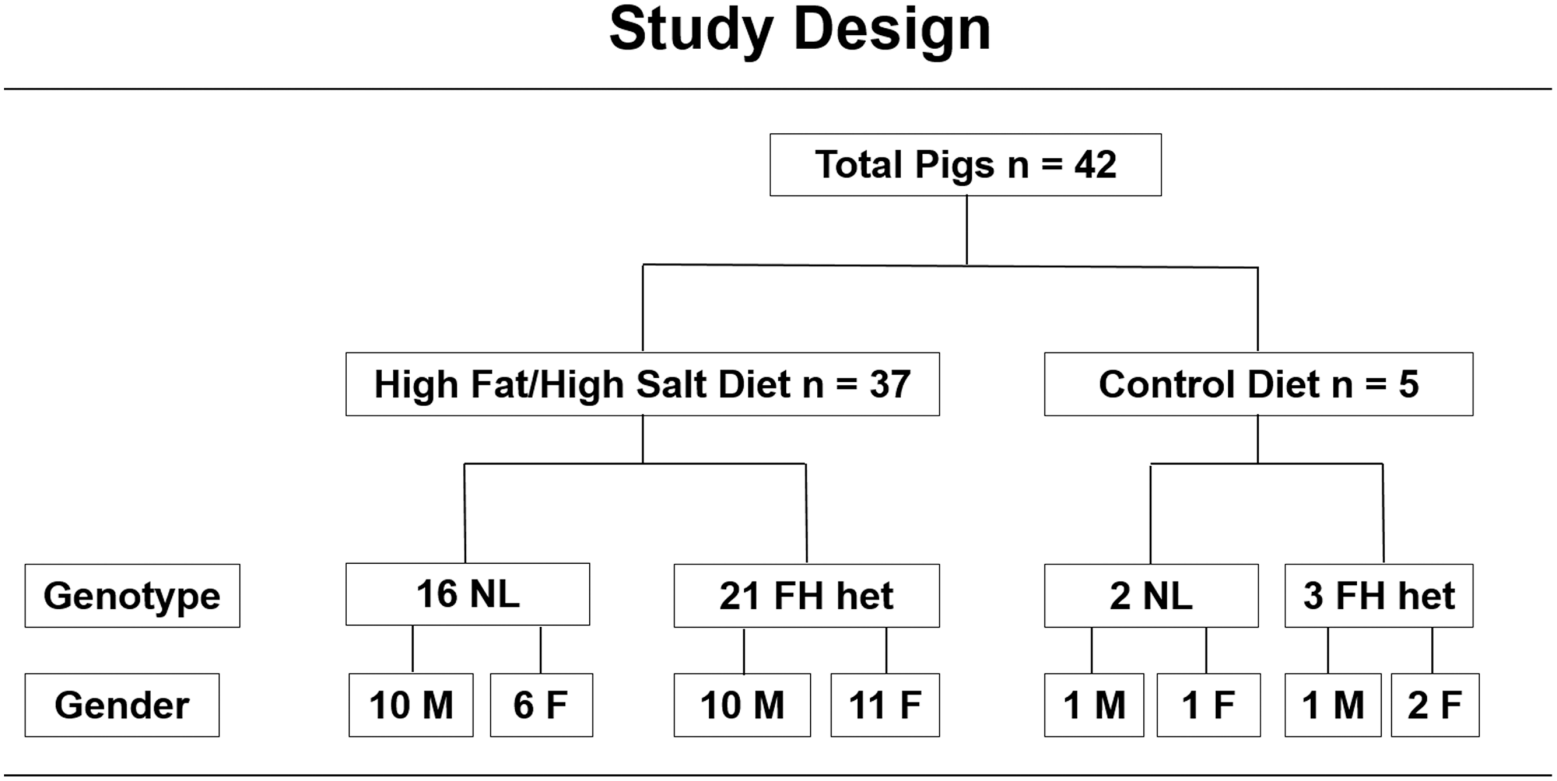
Study Design. The 42 pigs entered into this study are listed according to the diet they were fed, genotype (NL = normal, FH het = FH heterozygous) and gender.

All pigs were produced and maintained in the same environmental conditions at the Francis Owen Blood Research Laboratory at the University of North Carolina at Chapel Hill. The background was Spotted Poland/China and Yorkshire crosses. Male and female pigs from the following two genotypes were used: (1) normocholesterolemic or (2) heterozygous familial hypercholesterolemic (FH) that have a recessive inheritance pattern.[19–21] Both genotypes of pigs are normocholesterolemic at baseline and only exhibit hypercholesterolemia when fed a high fat diet. In addition to being identified by normal cholesterol levels, FH heterozygotes are also identified from litters produced by known sires and dams with normal LDL receptor sequences or carrying the FH mutation, a missense mutation in a single base pair (C_253_➔ T_253_) of the LDL receptor that results in an arginine_94_ to cysteine_94_ mutation, located in the region that corresponds to exon 4 in the human ligand binding domain.[19–21] Pigs were maintained in agricultural style Hog Slats Inc buildings with ambient light and dark cycles, free access to water, with a temperature range of 55 to 85 degrees F. All pigs were checked daily for food consumption and general health issues. The UNC Division of Laboratory Animal Medicine provided regular veterinarian oversight. Forty-two pigs were entered into the year long study as they became available (Fig 1). Thirty-seven pigs were fed a high fat/high NaCL diet for one year: 16 normal (10 males, 6 females) and 21 heterozygous FH (10 males, 11 females). Five pigs were fed regular pig chow: 2 normal (1 male, 1 female) and 3 heterozygous FH (1 male, 2 females). The mean age at study entry was 3.2 ± 1.6 years.

### Ethics Statement

All pigs were handled in strict accordance with the USDA regulations and the standards described in the 2010 Guide for the Care and Use of Laboratory Animals 8^th^ edition (http://grants.nih.gov/grants/olaw/Guide-for-the-Care-and-Use-of-Laboratory-Animals.pdf). All procedures and protocols were in accordance with institutional guidelines and approved by the Institutional Animal Care and Use Committee at the University of North Carolina in Chapel Hill (Animal Subject Assurance #A3410-01). When necessary, ketamine and acepromazine were given for sedation. See also PONE-D-15-01878R1 NC3Rs ARRIVE Guidelines Checklist Final in S1 File.

### High Fat/High NaCl Diet and Regular Pig Chow

The high fat /high NaCl diet consisted of pig chow (5NP8 Wayne 15% Pig & Sow Pellets, Granville Milling, Granville NC) supplemented with 1% cholesterol, 20% beef tallow, and 0.75% cholate by weight.[22, 23] The NaCl content of the diets was measured (Eurofins Scientific Inc, Des Moines, IA) and 5NP8 provided 0.35% or 8 grams/day as recommended[24] and the high fat diet provided 0.55% or 12.5 grams/day, a 56% increase. The total calories in the high fat/high NaCl diet were distributed as: 43% fat, 12.5% protein, and 44.5% carbohydrate. The 5NP8 was fed to the 5 control pigs as the source of regular pig chow. All pigs were fed in the morning once per day in their pen to be able to monitor and confirm food consumption.

### Sampling Protocol during Year Long Study

Weight, backfat, blood pressure, total and LDL and HDL cholesterol, oxLDL, fructosamine, aldosterone, triglycerides, Bergman Frequently Sampled Intravenous Glucose Tolerance Test (FSIVGTT), and serum and plasma inflammatory markers (TNF-alpha, IL-6, PAI-1, CRP) were obtained at baseline (BL), 3, 6, and 12 months of the year-long study. Serum glucose was measured monthly while the pig was fully conscious in addition to when it was sedated for the [25] FSIVGTT. Serum chemistry panels were obtained monthly to monitor general health status and processed by a commercial veterinary laboratory (Antech, Cary NC). At study termination all pigs were euthanized with an overdose of pentobarbital (6 grains/10 lbs. iv.) and tissue samples for morphometry were collected from all three coronary arteries and the abdominal aorta.

### Bergman Frequently Sampled Intravenous Glucose Tolerance Test (FSIVGTT)

Each pig was sedated with ketamine 4–6 mg/kg intramuscular (IM) and acepromazine 0.1–0.3 mg/kg IM. Two intravenous catheters were placed, one for sampling and one for infusing glucose and insulin. A bolus of glucose (0.3 gm/kg IV) was administered as a 50% solution over ~5 to 10 min. Serum was prepared from blood samples obtained at -15, -10, -5, -1, 2, 3, 4, 5, 6, 8, 10, 14, and 19 minutes to measure insulin and glucose concentrations. At 20 minutes, an insulin bolus (0.03U/kg IV, Novolin R Human Insulin Regular, Novo Nordisk) was injected and blood samples for insulin and glucose concentrations were collected at 22, 25, 30, 40, 50, 70, 100, 140, and 180 minutes. The data were analyzed by the Bergman method to calculate an insulin sensitivity index (S_i_) using MINMOD Millennium version 6.02.[26] This method has been widely used to estimate insulin sensitivity in humans and pigs.[25, 27–32]

### Measurement of Body Weight, Backfat, and Arterial Blood Pressure

Pigs were weighed on a FlexWeigh scale (model LPF-4824) with a digital readout (Velcon/FlexWeigh Model 5). Backfat has been used as an index of total body fat in pigs[33–38] and was measured by ultrasound over the last rib with the pig standing (Fukuda Denshi, model UF750XT). The intra-animal variability was < 7%. Arterial blood pressure was measured in the tail of conscious pigs (Veterinary Blood Pressure Monitor, model 9301V, CAS Medical Systems, Inc).[39–41] The results are the mean of 5 measurements taken over 5 min.

### Total and LDL and HDL Cholesterol, Triglycerides, Insulin, and Glucose

Serum cholesterol was measured with the Cholesterol E kit (Wako, Richmond, VA) that utilizes an enzymatic colorimetric method for quantitative determination of total cholesterol in serum. LDL and HDL cholesterol were measured by colorimetric endpoint reactions using the Liquid Direct LDL and HDL Cholesterol Kits, respectively (Amresco, Solon, OH). Reactions were performed according to the manufacturer’s instructions modified for a 96 well plate and optical density was read using a Vmax kinetic microtiter plate reader (Molecular Devices). Triglycerides were measured by an enzymatic method using triglyceride reagent (Sigma Aldrich). Serum insulin was measured by a solid phase RIA (ICN, lower unit of detection is 2 μU/ml). The intraassay variability was 4% and the interassay variability was 6%. Serum glucose was measured with a 2300 STAT PLUS (YSI, Yellow Springs, Ohio).

### Inflammatory Markers, oxLDL, Fructosamine, and Aldosterone

Pig specific ELISAs were used according to the manufacturer’s instructions to measure TNF-alpha (Biosource),[42] IL-6 (R&D Systems), PAI-1 (Molecular Innovations, Southfield, MI), [43] CRP (Pig CRP, Tri-Delta Diagnostics), and oxLDL (Mercodia, Uppsala, Sweden).[44–46] Fructosamine was measured in serum by a colorimetric endpoint reaction according to the manufacturer’s instructions (Raichem, San Diego, CA). Aldosterone was measured by enzyme immunoassay with a commercially available kit (Aldosterone EIA, Alpco Diagnostics, Salem, NH) using serum samples that were ether extracted, dried and reconstituted in sample buffer.

### Morphometry of Coronary and Abdominal Aortic Atherosclerosis

Coronary artery and aortic atherosclerosis were measured as previously described.[23, 47] Digital, calibrated images of all coronary and aortic sections were made by scanning the histological sections using the Aperio ScanScope and these images were measured using Aperio ImageScope software. Coronary artery atherosclerosis histomorphometry measurements include medial area, intimal area, percent stenosis and intimal area as percent medial area.[23, 47] Abdominal aortic atherosclerosis measurements include histomorphometry (i.e., medial area, intimal area, and intimal area as percent medial area) as well as en face morphometry measurements of the total area, lesion area and percent surface area with raised lesions.[23, 47] Coronary and aortic atherosclerosis was measured by at least two blinded observers using the same microscope, computer, and software. The inter- and intra- observer variability was between 4 and 7%.

### Definition of Severe and Diffuse Distal Coronary Artery Atherosclerosis

Severe coronary atherosclerosis was defined as mean intimal area as a percent of medial area of ≥ 200% in at least two coronary artery cross sections in the proximal or distal half. Diffuse distal coronary atherosclerosis was defined as a mean intimal area as a percent of medial area of ≥ 150% over at least three coronary artery cross sections separated by 2 cm in the distal half of the coronary artery.

### Total Internal Elastic Lamina (IEL) Area as an Assessment of Coronary Artery Remodeling

The total area contained within the IEL has been used to assess coronary arteries for remodeling during atherogenesis in humans and pigs.[5, 48, 49] Thus, the total IEL area for all three coronary arteries from the three groups of pigs was measured as described,[23, 47] and the results were expressed in mm^2^ for each of the three coronary arteries from all pigs.

### Percent of Coronary Artery Cross Sections with Fibrous Caps and Measurement of Fibrous Cap Thickness

The presence or absence of a fibrous cap was determined from visual inspection of all sections of each coronary artery from all pigs. The results are expressed as the percent of cross sections with fibrous caps. Maximal fibrous cap thickness was measured on the cross section with the greatest percent stenosis for a given coronary artery and is expressed in mm.

### Biostatistical Analyses

For all groups, descriptive statistics are reported as means and SD for weight, backfat, blood pressure, triglycerides, total and LDL and HDL cholesterol, oxLDL, fructosamine, aldosterone, inflammatory markers, insulin, glucose, Bergman S_i_ values, coronary and aortic atherosclerosis measurements. The coronary intimal area as a percent of medial area was used as the primary measure of atherosclerotic severity. The Wilcoxon rank sum statistic was used as the primary method for pairwise comparisons between severe and moderate atherosclerotic groups. Parametric repeated measures ANCOVA models with group, time, and baseline as explanatory factors were used as the primary method of finding association of logarithms of oxLDL, fructosamine, and aldosterone with atherosclerotic severity. In some of the models, atherosclerotic severity is dichotomous. Other models manage atherosclerotic severity as continuous, using a function of distal coronary arteries and intimal area as medial percentage. For blood pressure, total and LDL and HDL cholesterol, and triglycerides, Wilcoxon rank sum statistics for pairwise comparisons between groups and Wilcoxon signed ranks statistics for comparisons within groups were used with parametric repeated measures ANCOVA models in mutually supportive ways and with comparable results. The ANCOVA models additionally included gender and/or genotype as needed to account for their associations. A p-value less than 0.05 was the criterion for identifying a change within groups, a difference between groups, or an association of interest for future investigation.

## Results

The detailed results of the atherosclerosis data are presented first followed by the metabolic parameters that were monitored during the year long study to determine which ones were associated with the development of either the severe and diffuse distal versus moderate coronary atherosclerosis phenotype. Additional analyses that identified associations with p<0.05 for gender and genotype (i.e., normocholesterolemic and FH heterozygous) are listed.

### Severity of Coronary Artery Atherosclerosis

**Fig 2:**
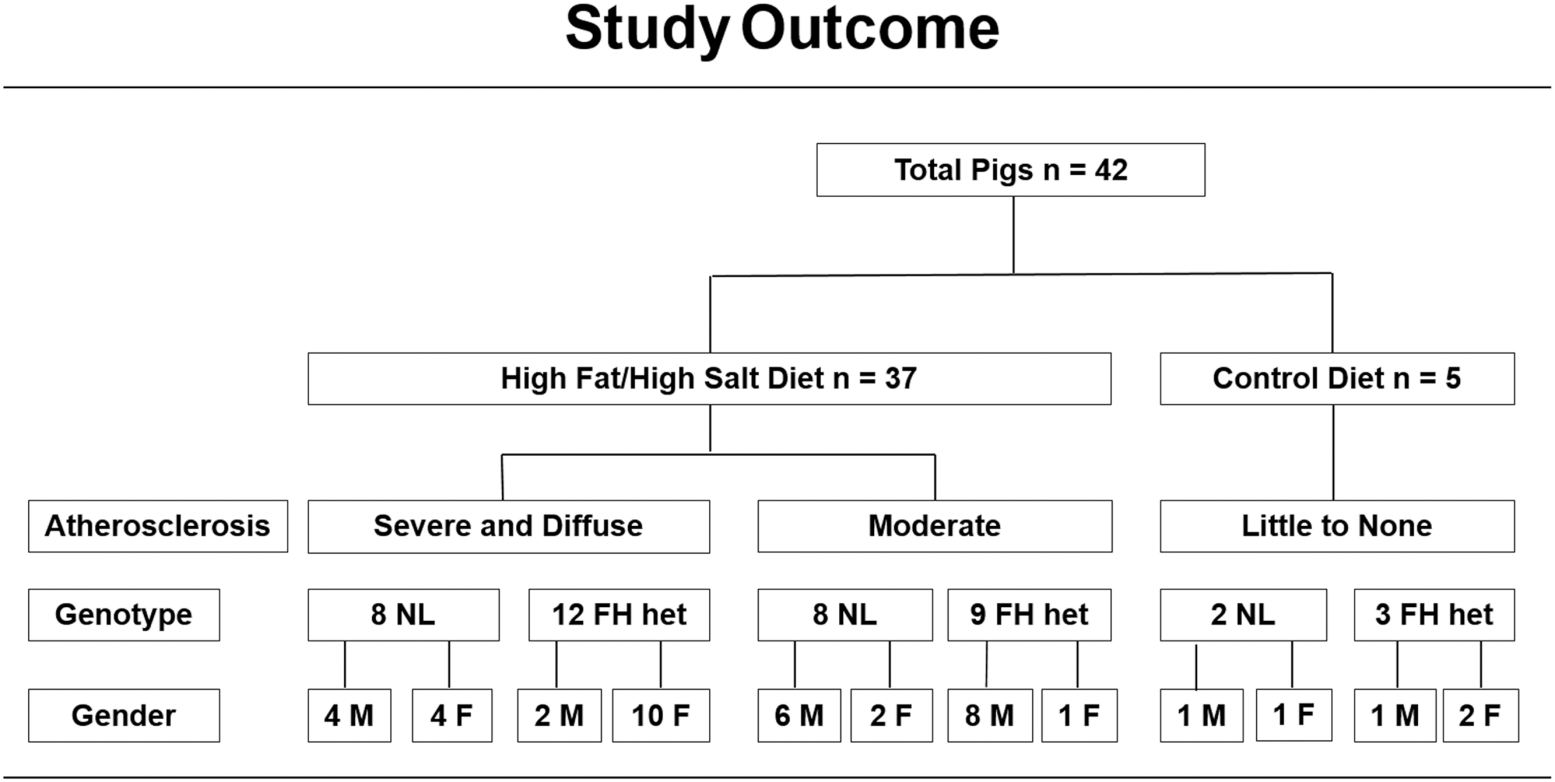
Study Outcome. The 42 pigs are described as in Fig 1 and grouped by atherosclerosis phenotype that developed during the 12-month study.

**Fig 3:**
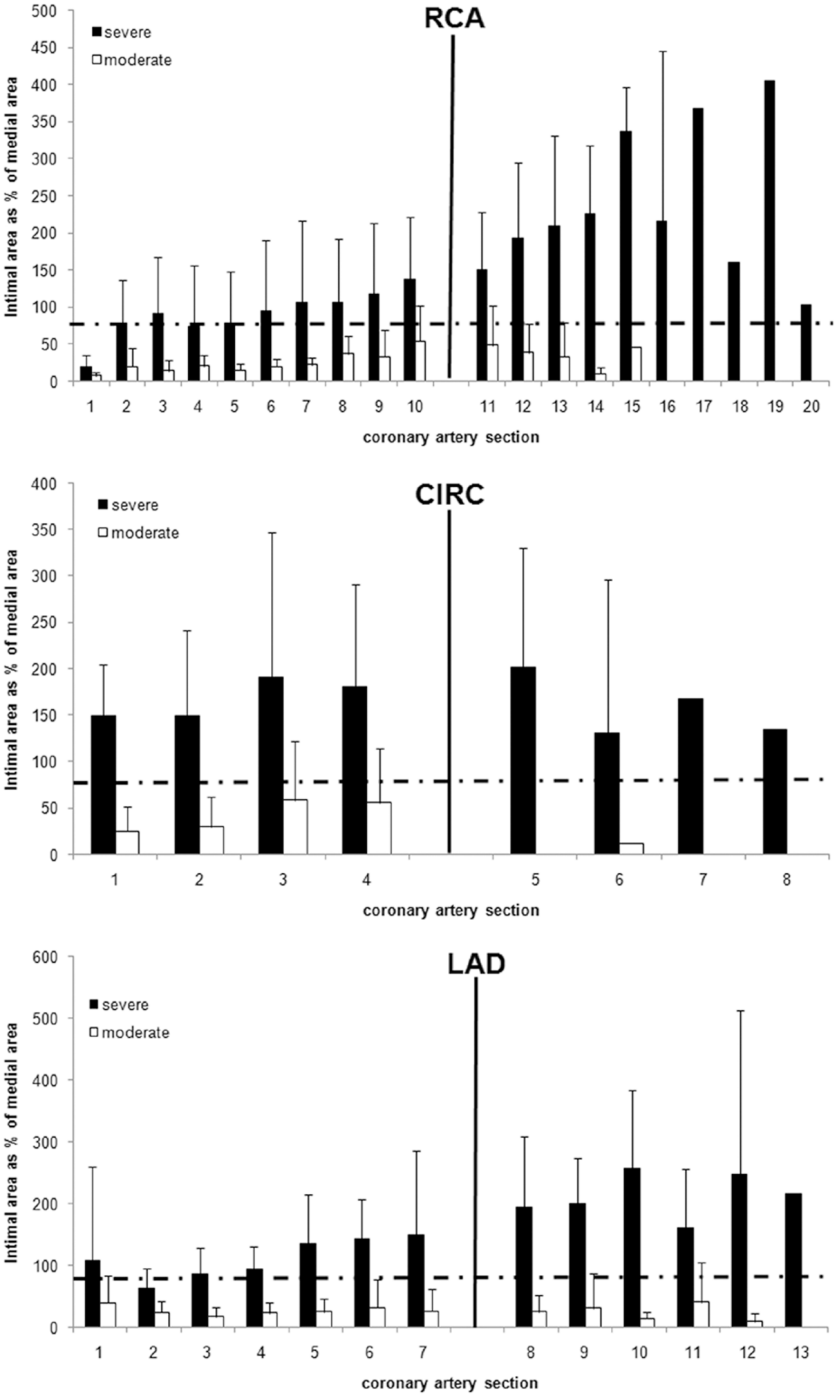
Intimal Area as % Medial Area from Serial Coronary Artery Sections. The mean ± SD for intimal area as % medial area of all sections from all three coronary arteries are shown by severe and diffuse (black), moderate (white), or control (grey) groups. Sections were taken at 1 cm intervals. The solid black vertical line indicates the division between the proximal and distal halves of each coronary artery. The horizontal black line at 200% indicates the criteria for severe coronary atherosclerosis. The hashed line at 150% indicates diffuse and distal coronary atherosclerosis when it occurs over 3 sections (i.e., 2 cm). Using these criteria, pigs in the severe atherosclerosis group also exhibited diffuse and distal coronary atherosclerosis in all three coronary arteries.

**Fig 4:**
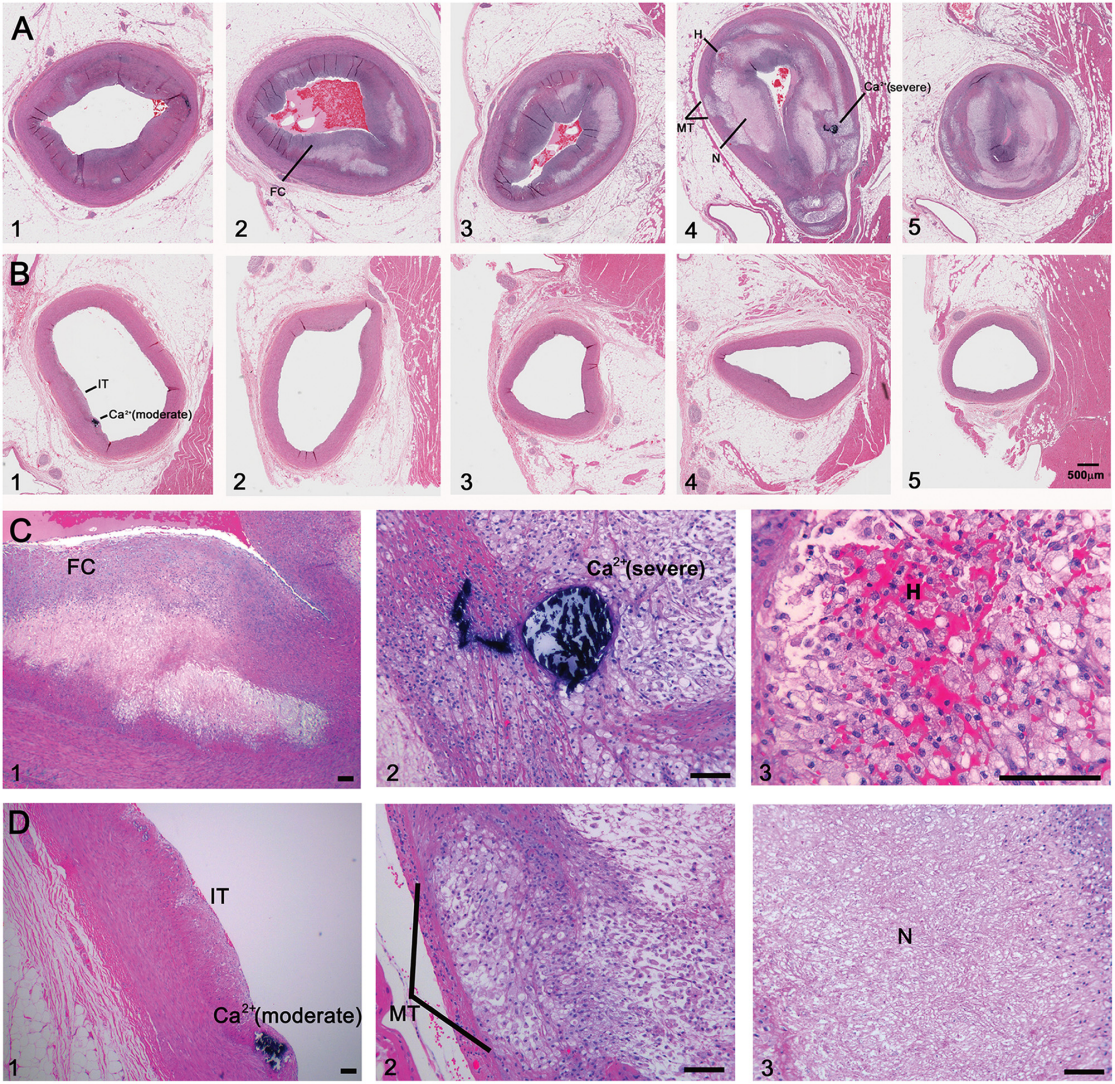
Coronary Artery Atherosclerosis in Insulin Resistant Pigs. Panels A1 to A5: Representative serial sections from the proximal to distal left anterior descending coronary artery are shown from an IR pig with severe and diffuse distal coronary atherosclerosis. The mean intimal area as a percent of medial area for all three coronary arteries for this pig was 203.6% proximally and 338.5% distally. Panels B1 to B5: In contrast, a pig with moderate coronary atherosclerosis had a mean intimal area as a percent of medial area for all three coronary arteries of 11.3% proximally and 5.8% distally. Features of coronary atherosclerosis in rows A and B are shown at higher magnification in rows C and D, respectively: intimal thickening (IT) and calcification in a pig with moderate atherosclerosis (Ca^2+^ (moderate), Panels B1 and D1), calcification in a pig with severe atherosclerosis (Ca^2+^ (severe), Panels A4 and C2), fibrous cap (FC, Panel A2 and C1), hemorrhage into the plaque (H, Panels A4 and C3), medial thinning (MT, Panels A4 and D2), necrosis (N, Panels A4 and D3). (Hematoxylin and Eosin, mag bar = 500 μm is shown in B5 for rows A and B; mag bar = 10 μm in all panels in rows C and D).

At the end of the study, 20 pigs (6 males and 14 females) fed the high fat/high NaCl diet were found to have met the criteria for both severe and diffuse distal coronary atherosclerosis (Figs 2–4). The other 17 pigs (14 males and 3 females) also fed the same high fat/high NaCl diet had substantially less or moderate coronary atherosclerosis. When the extent of coronary atherosclerosis was compared between the severe and moderate atherosclerosis groups for the proximal and distal halves of the coronary arteries, the severe group had much larger intimal area, percent stenosis, and intimal area as a percent medial area (Figs 3 and 4 and Table 1, p < 0.001). In a logistic regression model that controlled for gender, severe and diffuse coronary atherosclerosis was associated with female pigs (p = 0.003). Such a model that controlled for genotype instead of gender indicated no association of normal versus heterozygous FH genotype with severity of atherosclerosis (p = 0.963). The 5 pigs fed regular, low fat pig chow had little to no detectible coronary atherosclerosis (Table 1).

**Table 1:** Proximal and Distal Coronary Artery Atherosclerosis Morphometry.

	Proximal Coronary Arteries				Distal Coronary Arteries			
	Medial area (mm^2^)	Intimal area (mm^2^)	% Stenosis	Intimal area as % medial area	Medial area (mm^2^)	Intimal area (mm^2^)	% Stenosis	Intimal area as % medial area
**Severe and Diffuse Atherosclerosis** (n = 20)								
Mean	6.4	7.1	57.8	142.6	3.0	5.6	68.5	210.4
± SD	1.5	3.2	18.6	69.6	1.1	2.4	21.2	90.7
**Moderate Atherosclerosis** (n = 17)								
Mean	6.4	1.8	24.3	30.4	2.9	1.6	33.3	52.0
± SD	2.1	1.3	15.6	18.5	1.3	1.4	24.2	38.9
**Control** (n = 5)								
Mean	4.2	0.5	6.5	12.8	1.7	0.2	3.7	10.1
± SD	0.9	0.1	1.5	2.9	0.3	0.1	1.3	4.5
p*								
Severe vs Moderate	0.775	< 0.001	< 0.001	< 0.001	0.478	< 0.001	< 0.001	< 0.001
Severe vs Control	0.002	< 0.001	< 0.001	< 0.001	0.001	< 0.001	< 0.001	< 0.001
Moderate vs Control	0.031	0.011	0.006	0.039	0.071	< 0.001	< 0.001	0.006

* Wilcoxon rank sum statistic for differences between groups.

### Coronary Artery Remodeling Assessed by Total IEL Area

The severe atherosclerosis group had substantially larger total IEL areas when compared to the moderate (p < 0.001) or control groups (p<0.035) for all three coronary arteries (Table 2). The data are consistent with a greater degree of positive remodeling.[5, 48, 49] Also, there was no evidence of pairwise vessel to vessel differences in the total IEL area when comparing the three coronary arteries within the three groups of pigs (p > 0.1) except for the right versus circumflex coronary artery comparison within the moderate atherosclerosis group (p = 0.044).

**Table 2:** Mean Coronary Artery Total IEL Area (mm^2^), Percent of Coronary Artery Cross Sections Containing Fibrous Caps (FC), and Fibrous Cap Thickness (mm) in All 3 Coronary Arteries.

	Right			Circumflex			Left Anterior Descending		
	Total IEL Area (mm^2^)	% with FC*	Fibrous Cap (mm)	Total IEL Area (mm^2^)	% with FC*	Fibrous Cap (mm)	Total IEL Area (mm^2^)	% with FC*	Fibrous Cap (mm)
**Severe and Diffuse Atherosclerosis** (n = 20)									
Mean	10.1	81.6	0.472	9.9	88.8	0.521	9.8	83.9	0.377
± SD	2.1	16.4	0.184	3.1	24.2	0.277	2.1	17.0	0.139
**Moderate Atherosclerosis** (n = 17)									
Mean	6.2	26.2	0.405	5.0	41.3	0.482	6.0	32.2	0.346
± SD	1.7	22.5	0.194	2.4	41.9	0.208	2.3	37.1	0.124
**Control** (n = 5)									
Mean	6.1	1.3	0.510	6.6	0	N/A	6.4	0	N/A
± SD	1.1	3.0	0.000	1.7	0	N/A	0.6	0	N/A
**p** ^**†**^									
Severe vs Moderate	< 0.001	< 0.001	0.161	< 0.001	< 0.001	0.914	< 0.001	< 0.001	0.470
Severe vs Control	< 0.001	< 0.001	N/A	0.035	< 0.001	N/A	< 0.001	< 0.001	N/A
Moderate vs Control	1.00	< 0.001	N/A	0.164	< 0.001	N/A	0.820	< 0.001	N/A

*percent of coronary artery sections containing fibrous caps (FC),

^†^ Wilcoxon rank sum statistic for differences between groups,

N/A is not applicable,

### Percent of Coronary Artery Cross Sections with Fibrous Caps and Fibrous Cap Thickness

The percent of coronary artery cross sections containing fibrous caps was much greater in the severe and diffuse atherosclerosis group for all three coronaries (Table 2, p < 0.001). There was no evidence among animals with detectible fibrous caps of differences between the mean fibrous cap thicknesses for the severe and moderate groups (p > 0.1). Only one of the five control pigs had a very small fibrous cap lesion and that lesion was located in the right coronary artery of that pig.

### Abdominal Aortic Atherosclerosis

The 20 pigs that met the criteria for severe and diffuse distal coronary atherosclerosis had much more abdominal aortic atherosclerosis than the 17 pigs with moderate coronary atherosclerosis (Table A in S1 File for intimal area and intimal area as % medial area (p ≤ 0.004) and Table B in S1 File for en face measurement of % surface area with raised lesions, p ≤ 0.002). The 5 control pigs had raised lesions in the abdominal aorta (range was 2.9–28.6% of total surface area) that were much smaller than the other two groups (p ≤ 0.001). The intimal area as percent medial area was also much smaller in the control pigs (Table A in S1 File, p ≤ 0.04).

### Change in Fasting Glucose, Insulin, and Insulin Sensitivity by Bergman FSIVGTT during the Year Long Study

Throughout the study, changes in fasting glucose values in conscious pigs in any group were relatively small (Table 3 shows quarterly glucose values only, monthly values not shown). In contrast, when the pigs were sedated for the Bergman FSIVGTT, the glucose values were higher, possibly due to the stress of the procedure or the sedatives used. Differences between the groups at each time point were relatively small as were change in any group over time (p ≥ 0.05, Table 3).[50, 51]

**Table 3:** Fasting Glucose Values.

	Fasting Glucose (mg/dl) conscious				Fasting Glucose (mg/dl) sedated			
	Baseline	3 month	6 month	12 month	Baseline	3 month	6 month	12 month
**Severe and Diffuse Atherosclerosis** (n = 20)								
Mean	73	78	75	77	112	125	132	136
± SD	7	11	7	6	36	34	41	39
p*		0.054	0.163	0.050		0.091	0.092	0.062
**Moderate Atherosclerosis** (n = 17)								
Mean	75	73	76	75	114	113	114	121
± SD	7	9	6	7	23	21	29	28
p*		0.377	0.875	0.704		0.882	0.924	0.162
**Control (n = 5)**								
Mean	76	71	69	72	137	110	117	130
± SD	5	6	6	4	25	24	22	58
p*		0.248	0.121	0.516		0.067	0.251	0.821
p^†^								
Severe vs Moderate	0.263	0.136	0.807	0.476	0.661	0.254	0.156	0.247
Severe vs Control	0.235	0.116	0.067	0.154	0.086	0.371	0.530	0.265
Moderate vs Control	0.985	0.581	0.058	0.254	0.071	0.543	0.648	0.548

*Wilcoxon signed rank statistic for change from baseline within group.

^†^Wilcoxon rank sum statistic for differences between groups.

Fasting insulin values measured during the Bergman FSIVGTT were higher at 3 (Table 4, p < 0.001), 6 (p < 0.001), and 12 (p < 0.001) months when compared to baseline values for the severe and diffuse coronary atherosclerosis group and at 12 months in the moderate group (p = 0.006). The control group showed little or no evidence of change (p > 0.05, Table 4).

**Table 4:** Fasting Insulin Levels and Bergman Si Values.

	Fasting Insulin (μU/ml)—sedated				Bergman Si			
	Baseline	3 month	6 month	12 month	Baseline	3 month	6 month	12 month
**Severe and Diffuse Atherosclerosis** (n = 20)								
Mean	11.8	20.4	27.2	27.2	4.1	3.9	3.7	3.6
± SD	5.3	10.0	20.0	12.5	0.3	0.3	0.4	0.4
p*		< 0.001	< 0.001	< 0.001		< 0.001	< 0.001	< 0.001
**Moderate Atherosclerosis** (n = 17)								
Mean	13.0	17.3	16.7	18.8	4.0	3.7	3.7	3.5
± SD	10.0	9.1	10.0	8.7	0.4	0.4	0.4	0.4
p*		0.150	0.203	0.006		0.005	< 0.001	< 0.001
**Control (n = 5)**								
Mean	10.4	16.7	19.0	16.6	4.0	4.0	3.9	3.7
± SD	5.4	11.0	7.8	11.4	0.2	0.2	0.2	0.3
p*		0.253	0.067	0.378		1.000	0.033	0.058
p^†^								
Severe vs Moderate	0.775	0.266	0.127	0.056	0.103	0.312	0.282	0.581
Severe vs Control	0.530	0.408	0.818	0.088	0.310	0.205	0.179	0.584
Moderate vs Control	0.880	0.581	0.401	0.842	0.555	0.211	0.287	0.198

*Wilcoxon signed rank statistic for change from baseline within group.

^†^Wilcoxon rank sum statistic for differences between groups.

Analyses of fasting, 1 and 2 hour post prandial glucose and insulin levels in a subset of conscious pigs (n = 13 in the severe group, n = 6 in the moderate group, and n = 5 in the control group), showed similar trends (Tables F and G in S1 File).

The decreasing mean S_i_ values in the severe and moderate atherosclerosis groups at 3, 6 and 12 months were consistent with increased insulin resistance (p <0.001 for the severe and diffuse atherosclerosis group, and p ≤ 0.005 for the moderate atherosclerosis group compared to baseline, respectively). Likewise, the control pigs exhibited decreases in insulin sensitivity at 6 months (p = 0.033) and nearly so at 12 months (p = 0.058). There was little or no evidence of differences in mean fasting glucose, insulin or S_i_ values (Table 4) during the Bergman FSIVGTT between pairs of the three groups at any timepoint (Tables 3 and 4, p > 0.05).

### Change in Serum Fructosamine Levels during the Year Long Study

The log transformed serum fructosamine levels were similar at baseline for all three groups (ANOVA, p = 0.06). Fructosamine increased substantially at 3, 6, and 12 months compared to baseline in the group that developed severe and diffuse atherosclerosis (p ≤ 0.017), but only at month 3 for the moderate group (Table 5, p < 0.001). There was no evidence of change in the control pigs. The serum fructosamine level was much higher at 3, 6, and 12 months in the severe and diffuse atherosclerosis group when compared to either the moderate atherosclerosis or control groups (p ≤ 0.031).

**Table 5:** Fasting Fructosamine Levels (μmol/L).

	Baseline	3 month	6 month	12 month
**Severe and Diffuse Atherosclerosis** (n = 20)				
Mean	163	192	184	173
± SD	18	26	17	21
p*		< 0.001	< 0.001	0.017
**Moderate Atherosclerosis** (n = 17)				
Mean	148	171	159	150
± SD	23	24	26	23
p*		< 0.001	0.111	0.818
**Control (n = 5)**				
Mean	150	155	154	149
± SD	19	23	19	10
p*		0.221	0.058	0.961
p^†^				
Severe vs Moderate	0.027	0.011	<0.001	0.004
Severe vs Control	0.153	0.006	0.003	0.031
Moderate vs Control	0.880	0.283	0.928	0.842

*Wilcoxon signed rank statistic for change from baseline within group.

^†^Wilcoxon rank sum statistic for differences between groups.

### Change in Weight, Backfat, and Blood Pressure during Year Long Study

The baseline, 3, 6, and 12 month weight and backfat values and blood pressure results are shown in Tables C and D in S1 File, respectively. Weight increased substantially over time compared to baseline in the moderate atherosclerosis group (p < 0.001) and in the severe group (p < 0.001). There was no evidence of differences between the severe and moderate groups for weight at study entry nor at any of the time points (p ≥ 0.4). Weight in the control group increased moderately over time (p <0.001 at month 12, Table C in S1 File). Backfat also increased substantially over time compared to baseline in the severe and diffuse atherosclerosis group (p ≤ 0.003) and in the moderate atherosclerosis group (p ≤ 0.005). There was no evidence of differences between the severe and moderate groups for backfat at study entry (p = 0.265) but there was ~ 1 cm more backfat at all three time points in the severe group (p ≤ 0.016). Back fat in the control group increased relatively minimally over time (p ≥ 0.070).

Blood pressure was in a range that would be considered mildly hypertensive in humans (142–160 / 93–108 mmHg) with little or no evidence of interpretable differences between the three groups (Table D in S1 File, p ≥ 0.10 for 19 of 24 cases). Blood pressure had little or no evidence for interpretable changes over time in any group (p≥ 0.10 for 15 of 18 cases).

### Total, LDL, and HDL Cholesterol and Triglyceride Levels

The mean total cholesterol values ranged from 77 to 110 mg/dl at baseline, but it was lower for the group that developed moderate atherosclerosis than for both the group that developed severe and diffuse atherosclerosis and the control group (Table 6, p ≤ 0.004). A similar pattern was seen at baseline for LDL and HDL cholesterol (Tables 6 and 7) where the mean value for the moderate group was substantially lower than severe or control, but the control and severe were not substantially different. Fasting triglycerides values had little to no differences between the three groups at study entry (Table 7). For the pigs fed the high fat/high NaCl diet, fasting total, LDL, and HDL cholesterol increased substantially at 3, 6, and 12 months relative to baseline values within severe and moderate groups (p < 0.001), but evidence of change for triglycerides was only applicable to the severe group. There were substantial differences between the moderate and severe groups at 3, 6, and 12 months for total and LDL cholesterol and triglycerides (p < 0.02), but HDL had no interpretable differences. Control pigs showed no essentially no or minimal changes in these measurements over the 12 month study and had much lower total cholesterol, HDL, and LDL values when compared to the pigs being fed the high fat/high NaCl diet (p > 0.05 for all but one value).

**Table 6:** Fasting Total and LDL Cholesterol.

	Total cholesterol (mg/dl)				LDL cholesterol (mg/dl)			
	Baseline	3 month	6 month	12 month	Baseline	3 month	6 month	12 month
**Severe and Diffuse Atherosclerosis** (n = 20)								
Mean	103	583	481	413	57	226	186	157
± SD	26	188	127	130	13	81	44	34
p*		<0.001	<0.001	<0.001		<0.001	<0.001	<0.001
**Moderate Atherosclerosis** (n = 17)								
Mean	77	416	366	320	44	172	143	131
± SD	20	107	163	74	14	38	50	27
p*		<0.001	<0.001	<0.001		<0.001	<0.001	<0.001
**Control (n = 5)**								
Mean	110	113	119	106	57	64	66	61
± SD	25	32	29	26	9	16	12	16
p*		0.922	0.132	0.465		0.133	0.027	0.335
p^†^								
Severe vs Moderate	0.002	0.003	0.021	0.015	0.005	0.017	0.009	0.016
Severe vs Control	0.564	<0.001	<0.001	<0.001	0.809	<0.001	<0.001	<0.001
Moderate vs Control	0.004	<0.001	0.001	<0.001	0.056	<0.001	0.001	<0.001

*Wilcoxon signed rank statistic for change from baseline within group.

^†^Wilcoxon rank sum statistic for differences between groups.

**Table 7:** Fasting HDL Cholesterol and Triglycerides.

	HDL cholesterol (mg/dl)				Triglycerides (mg/dl)			
	Baseline	3 month	6 month	12 month	Baseline	3 month	6 month	12 month
**Severe and Diffuse Atherosclerosis** (n = 20)								
Mean	40	81	71	65	36	47	43	52
± SD	8	29	13	15	29	28	25	39
p*		<0.001	<0.001	<0.001		0.013	0.235	0.035
**Moderate Atherosclerosis** (n = 17)								
Mean	34	82	77	78	21	25	25	21
± SD	11	28	28	19	6	12	13	11
p*		<0.001	<0.001	<0.001		0.475	0.662	0.463
**Control (n = 5)**								
Mean	44	48	48	47	29	45	45	36
± SD	6	6	8	7	12	21	30	15
p*		0.119	0.266	0.074		0.072	0.228	0.136
p^†^								
Severe vs Moderate	0.049	0.934	0.437	0.028	0.046	0.005	0.011	0.004
Severe vs Control	0.330	0.009	<0.001	0.024	0.830	0.986	0.988	0.667
Moderate vs Control	0.025	0.006	0.048	0.001	0.182	0.032	0.240	0.020

*Wilcoxon signed rank statistic for change from baseline within group.

^†^Wilcoxon rank sum statistic for differences between groups.

### Changes in Oxidized LDL

The mean oxLDL levels for the severe and diffuse atherosclerosis group at baseline, while similar to the moderate atherosclerosis group, was higher than the control group (Table 8, p = 0.015). The values increased substantially relative to the baseline at 3, 6, and 12 months in pigs fed the high fat/high NaCl diet (p ≤ 0.002) but not control pigs (p > 0.46). OxLDL was substantially higher in the severe atherosclerosis group at 3, 6 and 12 months compared to the moderate group and it was substantially lower for the control group (Table 8, p ≤ 0.002).

**Table 8:** Fasting oxLDL Levels (units/L).

	Baseline	3 month	6 month	12 month
**Severe and Diffuse Atherosclerosis** (n = 20)				
Mean	20.4	47.9	42.6	39.8
± SD	5.3	17.5	11.3	10.6
p*		<0.001	<0.001	0.002
**Moderate Atherosclerosis** (n = 17)				
Mean	18.7	31.5	31.3	29.8
± SD	4.8	7.1	8.2	7.0
p*		<0.001	<0.001	<0.001
**Control (n = 5)**				
Mean	13.6	12.6	14.1	13.5
± SD	4.7	3.1	4.3	3.7
p*		0.465	0.465	0.465
p^†^				
Severe vs Moderate	0.410	<0.001	<0.001	0.002
Severe vs Control	0.015	<0.001	<0.001	<0.001
Moderate vs Control	0.088	<0.001	<0.001	<0.001

*Wilcoxon signed rank statistic for change from baseline within group.

^†^Wilcoxon rank sum statistic for differences between groups.

### Changes in Aldosterone

The mean aldosterone levels were similar for all three groups at baseline (p>0.9, Table E in S1 File). The values did not increase in the control group or group with moderate coronary atherosclerosis whereas in the group with severe disease there was a moderate increase above baseline values at 6 and 12 months (p ≤ 0.042). When controlling for gender, however, the changes in aldosterone were not associated with severe and diffuse atherosclerosis (p > 0.15). Serum potassium and sodium levels were assayed monthly and remained within the normal range (not shown).

### Change in Inflammatory Markers during Year Long Study

The inflammatory markers (TNF-alpha, IL-6, PAI-1, CRP) did not differ between the moderate and severe groups in 9 pigs with severe and diffuse coronary atherosclerosis when compared to 8 pigs with moderate disease (data not shown). Since these values did not appear to be robust markers of IR or atherosclerosis in this model, the assays were not performed in the remaining pigs.

### Adverse Events

During the year-long study, transient inappetance occurred in both the severe and diffuse (5/20) and moderate (6/17) groups but not the control group. In general, inappetance responded to feeding regular chow for a few days before restarting the high fat/high NaCL diet. If the pig had hemoccult positive stools, appropriate medications were given with the advice of the attending veterinarian (e.g., antacids and proton pump inhibitors). Two of the 6 pigs with inappetance in the moderate atherosclerosis group required blood transfusions for anemia due to gastrointestinal bleeding. In all cases, sampling was not interrupted by these events. One pig in the moderate atherosclerosis group was euthanized for persistent inappetance after 6 months on study. One pig in the severe atherosclerosis group died suddenly at age 10 months and, in addition to severe and diffuse coronary atherosclerosis, had a bladder infection at necropsy. Both of these animals are included in all analyses.

## Discussion

The study shows that 37 adult pigs fed a high fat/high NaCl diet exhibited a substantial increase in IR that was accompanied by weight gain, increased backfat, and elevations in total and LDL and HDL cholesterol levels. Those pigs that exhibited more severe as well as diffuse distal coronary artery and abdominal aortic atherosclerosis had substantially greater increases in fructosamine and oxLDL levels during the study compared to the pigs with moderate disease or the control pigs. Although all animals developed increased IR during the study as reflected in the decreasing S_i_ values, the severity of IR was not associated with the severity of atherosclerosis. This finding suggests that this degree of IR did not discriminate between animals that develop severe and diffuse vs moderate atherosclerosis in this model. Since none of the IR pigs had diabetes, it appears that severe and diffuse atherosclerosis can develop in response to a high fat/high NaCl diet in the absence of overt hyperglycemia but this severe atherosclerosis phenotype is associated with increased oxLDL and fructosamine levels consistent with increased protein glycation. These findings are also reinforced by the fact that the normal chow-fed controls also developed IR but had no substantial change in cholesterol, oxLDL, or fructosamine levels and little to no atherosclerosis.

### Insulin Resistance

The manner in which the hyperinsulinemic state or the loss of insulin sensitivity might lead to increased atherosclerosis remains poorly defined. Recently, several mechanisms that mediate insulin resistance in mice, pigs, and humans have been described.[52–58] It is not yet know if these mechanisms also mediate atherogenesis in addition to IR. Additionally the presence of IR is often associated with hypertension, lipoprotein abnormalities, and chronic kidney disease that further accentuate atherosclerosis risk.[59–64]

Although fully manifested diabetes mellitus in humans confers a greater risk for cardiovascular disease and other complications as compared to isolated IR without hyperglycemia, the presence of IR is a major independent risk factor with relative risk ratios between 2.2 and 2.7 when multifactorial linear regression analysis is utilized to assess risk.[2, 65, 66] Within this broad category of insulin resistance, there are several subgroups of patients with different metabolic abnormalities. Specifically some subjects have hyperlipidemia without overt diabetes, some have diabetes without hyperlipidemia, and both groups can have abnormalities in glucose dynamics and elevated levels of oxidized LDL as well as other glycated proteins such as HgbA1c and fructosamine.

Our IR pigs had elevated glucose levels during the Bergman FSIVGTT when sedated but not when fully conscious (Table 3). It is possible that the sedatives used in these pigs contributed to the glucose elevations (i.e., acepromazine and ketamine). Isoflurane has been shown to alter insulin sensitivity in pigs, possibly from direct drug effects as well as procedure-related stress.[50, 51] Importantly, since all S_i_ measurements were done with sedation, the observed reduction in S_i_ over 12 months is interpretable and was clearly progressive. Despite this increase in insulin resistance, however, there was no detectible association between the degree of changes in insulin sensitivity and the severity of atherosclerosis in the pigs in this study.

### Cholesterol Levels and Atherosclerosis Severity

All pigs fed the high fat/high NaCl diet exhibited hypercholesterolemia but the levels were higher in pigs that developed severe and diffuse atherosclerosis. This difference in total and LDL cholesterol levels accounts for some, but not all, of the observed differences between groups according to atherosclerosis severity. In mixed models that used a continuous measure of coronary artery atherosclerosis severity, controlling for gender, baseline values, and variations in total cholesterol over time, there are still substantial strengths of association between coronary artery atherosclerosis severity and oxLDL (p = 0.035) and fructosamine (p = 0.022) that are independent of the changes in total or LDL cholesterol. Such models also indicated an association between the variation of total cholesterol over time with fructosamine (p< 0.001) and oxLDL (p< 0.001). Thus, while there are associations of cholesterol levels and atherosclerosis severity, the more noteworthy issue for cholesterol was the extent to which it fully or partly explained the associations of atherosclerosis severity with increased levels of fructosamine and oxLDL.

### Fructosamine

Fructosamine is used as an index of mean blood glucose and is predictive of incident diabetes and microvascular complications in humans.[67] In pigs, fructosamine is also a reliable and important marker of mean blood glucose since pig red blood cells are relatively impermeable to glucose and thus hemoglobin A1c is not a reliable index of mean glucose concentrations in this species.[16, 68] Fructosamine is also a measure of protein glycation.[69] In our study, the fructosamine levels measured during the fasting state rose relative to baseline values in the group of animals that developed severe and diffuse coronary atherosclerosis but the levels were not elevated to a point that they would reflect meaningful and/or sustained hyperglycemia.

With regards to blood glucose levels, studies in humans have shown that stress-induced hyperglycemia correlates with subsequent development of diabetes and that the subjects who were predisposed to develop glucose intolerance during the FSIVGTT had higher fructosamine levels at baseline.[70] As mentioned above, the IR pigs appeared to exhibit stress-associated hyperglycemia during the Bergman FSIVGTT that may have accounted for some portion of the higher fructosamine levels. Fructosamine has also been shown to correlate with 2 hr postprandial glucose[71] and relatively short periods of hyperglycemia have been shown to increase protein glycation even if fasting glucose is not increased.[72] Presumably the increase in fructosamine in the IR pigs with severe and diffuse atherosclerosis reflects a modest increase in mean daily glucose levels, although periods of post prandial hyperglycemia of short duration followed by periods of normoglycemia as the sole cause cannot be excluded. Regardless of the cause, the degree of fructosamine increase was substantially greater in pigs with severe and diffuse atherosclerosis than the change that occurred in the group with moderate atherosclerosis or the control group. Therefore, it is possible that even a relatively modest change in fructosamine reflects a contribution of increased mean glucose values that would contribute to increased protein glycation and atherosclerosis development.

With regards to atherosclerosis, most reports have compared the degree of fructosamine change that occurs with impaired glucose tolerance to subjects with normal glucose tolerance and correlated this with a change in atherosclerotic risk. Studies in experimental animal models have reported increases in fructosamine over the range noted in our animals as being associated with biochemical changes that are believed to predispose to increase risk of atherosclerosis and some studies in humans with impaired glucose tolerance have demonstrated an increase in carotid intimal media thickening.[73] Although studies in which fructosamine changes alone, independent of changes in lipoproteins, predispose to increased coronary and aortic atherosclerosis have not been reported, there are studies wherein changes in advanced glycation end products or glycated albumin (another surrogate measurement of serum glycated protein concentrations) have been shown to predict atherosclerosis risk.[74–77] When measures of advanced glycation end products other than fructosamine or glycated albumin are utilized, there are also strong associations with the presence of advanced atherosclerosis.[78–80] Importantly, in a prospective study in adult humans without diabetes, higher HgbA1c was independently associated with progression of advanced coronary artery calcification.[81] Therefore, the combination of abnormal blood glucose dynamics that are not classified as overt diabetes and elevated concentrations of glycated proteins is associated with the development of advanced coronary atherosclerosis in humans and pigs.

### Oxidized LDL (oxLDL)

Increases in oxidized LDL in humans, pigs, and rodents are associated with increased atherosclerosis and reduction in oxLDL is associated with reduced atherosclerosis.[82–87] The mechanism by which these changes in oxLDL might modulate atherosclerosis severity cannot be completely separated from the changes induced by protein glycation since exposure of LDL to high glucose concentrations results in abnormal glycation as well as oxidation.[88] In humans, increased glycemic index is also associated with increased oxLDL when total LDL remains unchanged.[82, 89–91] The more modest increase in postprandial glucoses in subjects with impaired glucose tolerance can also lead to elevated oxLDL[92–95] and treatment of those subjects lowers oxLDL.[96–99] Also, reductions in fat mass, dietary fat, and blood glucose have been shown to be associated with decreases in oxLDL.[100–103] A few studies have reported increases in oxLDL in nondiabetic subjects with impaired glucose tolerance[104–106] and one reported a positive correlation between oxLDL in normal subjects who also exhibited higher fructosamine levels.[104] Moreover, even small increases in postprandial glucose that are within the normal range can lead to increases in oxidized LDL[82, 107] and small reductions in postprandial glucose in patients with type 2 diabetes also lead to decreased oxidized LDL.[82, 107] Since oxidized LDL was increased in the pigs that developed severe and diffuse atherosclerosis, it is possible that increases solely in their postprandial glucoses were sufficient to contribute to the elevation in oxLDL levels.

### Atherosclerosis Morphology in Insulin Resistance

Coronary artery atherosclerosis in the IR pigs showed fibrous cap formation, foam cells, medial thinning, plaque hemorrhage, necrosis, and calcification (Fig 4). All of these characteristics have been described in atherosclerotic plaques from IR and diabetic humans.[5, 7] Similar histological findings have been described in pigs given streptozotocin and fed a high fat diet.[16] An important characteristic is the presence of distal coronary atherosclerosis which has been a consistent finding in asymptomatic IR and diabetic patients screened by electron beam computed tomography and magnetic resonance imaging,[4, 8] clinically symptomatic IR patients by quantitative angiography[6] and in type 2 diabetic patients who died suddenly.[5] Thick fibrous caps (>64 μm) are more common in coronary atherosclerotic plaques of humans with type 2 diabetes who died suddenly.[5] In our study, the fibrous caps were more abundant in pigs with severe atherosclerosis than those with moderate atherosclerosis (Table 2).

Clinical studies have reported an increase in coronary artery remodeling in diabetic victims of sudden death.[5] The mean total IEL areas for serial sections in all 3 coronary arteries from IR pigs with severe atherosclerosis was larger than those with moderate atherosclerosis (Table 2). This finding is consistent with positive remodeling.[5, 48, 49] Thus, the coronary atherosclerotic plaques in the IR pigs with severe and diffuse disease also exhibited many of the phenotypic characteristics that are found in IR humans.

### Gender

Severe and diffuse atherosclerosis occurred more commonly in IR female pigs fed the high fat/high NaCl diet. One study conducted in nondiabetic female minipigs designed to develop metabolic syndrome found they became more insulin resistant and developed more atherogenic lipoprotein changes compared to males.[108] In insulin resistant humans, incident cardiovascular disease is more prevalent in women from many different ethnic groups including Caucasians, Latinos, American Indians, and several European populations.[109] Additionally increased levels of glycated proteins predicted mortality from coronary heart disease in non-diabetic women but not non-diabetic men.[74] The reason for this difference between genders is unknown. It is speculated that the difference may in part be explained by a higher burden of all risk factors for cardiovascular disease and a greater effect of high blood pressure and atherogenic dyslipidemia in women when compared to men.[110]

### Mechanism of glycation induced changes in atherosclerosis severity

Although our study was not designed to identify or measure the mechanism by which changes in oxLDL or glycated proteins would induce atherogenesis, the cell surface receptor RAGE is thought to be a key mediator.[111] RAGE is induced in blood vessels under these conditions and the soluble form of RAGE that is secreted is increased in patients with severe atherosclerosis and abnormal protein glycation. The mechanism by which these combinations of metabolic changes induce atherosclerosis has been proposed to be activation of macrophages that in turn further oxidize LDL. An additional proposed mechanism is the inhibition of autophagy. Specifically glycated albumin results in autophagy dysfunction in pancreatic beta cells leading to enhanced pancreatic cell death.[112] A reduction in the rate of autophagy induced by glycated proteins has also been implicated in pericyte dropout and the subsequent development of diabetic retinopathy.[113] Finally inhibition of autophagy has been shown to be important for mediating changes in endothelial cells that have been associated with atherosclerosis.[114] The results of our study support the hypothesis that elevations in glycated proteins in IR pigs could lead to more severe and diffuse atherosclerosis at least in part through inhibition of autophagy.

### Study Limitations

We chose to measure parameters that have been associated with increased atherosclerosis and insulin resistance in humans: blood pressure, body weight, body fat, hyperlipidemia, inflammatory markers, glycated proteins including fructosamine and oxLDL, and aldosterone. This study design addressed the extent to which each of these parameters, as measured at 4 time points, were associated with the severity of atherosclerosis that developed in pigs with comparable IR. The grouping of pigs by atherosclerosis severity was only possible after feeding the high fat diet for one year. As the investigators did not know which pigs had severe atherosclerosis until the end of the study, one can regard the investigators as being blinded. Likewise, evaluating these parameters for association with atherosclerosis severity could only be done after identifying the groups according to specific criteria that were defined *a priori*. Although possibly introducing some bias, this grouping is comparable to a ranking that classified somewhat more than half as severe and the others as moderate. We have additionally addressed this possibility by analyzing changes over time with appropriate and validated statistical methods.[115] We may have inadvertently omitted other parameters that could be associated with atherosclerosis severity that could also be analyzed in a similar fashion. We did not measure glycated proteins in tissues so we do not know the extent to which such measurements would or would not have correlated with the fructosamine and oxLDL levels measured in the circulation. The other limitation of our study is that it is purely hypothesis generating, and not hypothesis testing, as many pairwise comparisons were made.

## Conclusion

Our goal was to develop a useful animal model of insulin resistance that also develops coronary artery and aortic atherosclerosis. Several investigators have used pigs to study the impact of diabetes on atherogenesis, most of whom have used chemical induction of insulin-deficiency.[16, 18, 116, 117] In contrast, we used pigs that developed increased insulin resistance in association with weight gain, increased backfat, increased total and LDL and HDL cholesterol levels, and elevated blood pressure. Our model has some features of the metabolic syndrome and about half of the animals developed more severe and diffuse atherosclerosis. The metabolic variables that are associated with atherosclerosis severity are increases in fructosamine and oxidized LDL. Our study had a robust number of pigs and it would be reasonable expect to see a similar effect size in future studies. This animal model should be useful for identifying and studying mechanisms of disease that are suggested by these associations and for testing new therapeutic approaches designed to reduce or prevent the development of severe and diffuse atherosclerosis in insulin resistant subjects. Our data provide a rational basis for determining the number of animals required for such a mechanistic-based study to detect significant differences between treatment groups.

## Supporting Information

S1 File Tables A through G and PONE-D-15-01878R1 NC3Rs ARRIVE Guidelines Checklist Final. (DOCX)
