# Supplementary material for: Oxidized LDL and Fructosamine Associated with Severity of Coronary Artery Atherosclerosis in Insulin Resistant Pigs Fed a High Fat/High NaCl Diet
Source: PLoS One. 2015 Jul 6;10(7):e0132302. doi: 10.1371/journal.pone.0132302 (PMC4492503; doi:10.1371/journal.pone.0132302)

Supporting Information

Title: Oxidized LDL and Fructosamine Associated with Severity of Coronary Artery Atherosclerosis in Insulin Resistant Pigs Fed a High Fat/High NaCl Diet

Short Title: Severe and Diffuse Coronary Atherosclerosis in Insulin Resistant Pigs

Timothy C. Nichols^1,2*^, Elizabeth P. Merricks^1^, Dwight A. Bellinger^1^, Robin A. Raymer^1^,

Jing Yu^3^ Diana Lam^3^, Gary G. Koch^3^, Walker H. Busby Jr.^2^, David R. Clemmons^2^

^1^Department of Pathology and Laboratory Medicine, University of North Carolina at Chapel Hill, Chapel Hill, North Carolina, United States of America

^2^Department of Medicine, University of North Carolina at Chapel Hill, Chapel Hill, North Carolina, United States of America

^3^Department of Biostatistics, University of North Carolina at Chapel Hill, Chapel Hill, North Carolina, United States of America

Short running head: Coronary Atherosclerosis in Insulin Resistant Pigs

^*^Corresponding author

Email: [tnichols@med.unc.edu](mailto:tnichols@med.unc.edu) (TCN)

Keywords: Atherosclerosis; coronary artery disease; diffuse coronary atherosclerosis; coronary artery atherosclerosis severity; insulin resistance; diabetes mellitus; pigs; swine; glycated proteins; fructosamine; oxidized LDL; hypercholesterolemia; hyperlipidemia

**Table A. Abdominal Aortic Atherosclerosis Histomorphometry**

|  |  | Medial area (mm^2^) | Intimal area (mm^2^) | Intimal area as % medial area |
| --- | --- | --- | --- | --- |
|  |  |  |  |  |
| **Severe and Diffuse Atherosclerosis** (n = 20) | | | |  |
|  |  |  |  |  |
| Mean |  | 31.9 | 7.2 | 23.6 |
| ± SD |  | 7.2 | 2.9 | 10.8 |
|  |  |  |  |  |
| **Moderate Atherosclerosis** ( n = 17) | | |  |  |
|  |  |  |  |  |
| Mean |  | 34.3 | 4.4 | 12.0 |
| ± SD |  | 10.5 | 4.9 | 11.7 |
|  |  |  |  |  |
| **Control** (n=5) | | |  |  |
|  |  |  |  |  |
| Mean |  | 26.0 | 0.9 | 3.3 |
| ± SD |  | 3.5 | 0.8 | 3.4 |
|  |  |  |  |  |
|  |  |  |  |  |
| p* |  |  |  |  |
| Severe vs Moderate |  | 0.460 | 0.004 | 0.002 |
| Severe vs Control |  | 0.071 | < 0.001 | < 0.001 |
| Moderate vs Control |  | 0.048 | 0.019 | 0.039 |
|  |  |  |  |  |

* Wilcoxon rank sum statistic for differences between groups

**Table B. Abdominal Aortic Atherosclerosis En Face Morphometry**

|  |  | Total  Aortic surface area (cm^2^) | Area with raised lesion (cm^2^) | | % aortic surface with raised lesions |
| --- | --- | --- | --- | --- | --- |
|  | | | |  | |
| **Severe and Diffuse Atherosclerosis** (n = 20) | | | | |  |
|  |  |  |  | |  |
| Mean |  | 30.5 | 19.0 | | 61.5 |
| ± SD |  | 5.9 | 6.2 | | 14.0 |
|  |  |  |  | |  |
| **Moderate Atherosclerosis** ( n = 17) | | |  | |  |
|  |  |  |  | |  |
| Mean |  | 30.8 | 13.8 | | 42.6 |
| ± SD |  | 7.0 | 8.6 | | 19.6 |
|  |  |  |  | |  |
| **Control** (n=5) | | |  | |  |
|  |  |  |  | |  |
| Mean |  | 25.2 | 3.4 | | 14.1 |
| ± SD |  | 6.8 | 2.8 | | 11.0 |
|  |  |  |  | |  |
|  |  |  |  | |  |
| p* |  |  |  | |  |
| Severe vs Moderate |  | 0.792 | 0.026 | | 0.002 |
| Severe vs Control |  | 0.188 | < 0.001 | | < 0.001 |
| Moderate vs Control |  | 0.085 | 0.002 | | 0.001 |
|  |  |  |  | |  |

* Wilcoxon rank sum statistic for differences between groups

**Table C. Weight and Backfat**

|  |  | **Weight (lb)** | | | |  | **Backfat (cm)** | | | |
| --- | --- | --- | --- | --- | --- | --- | --- | --- | --- | --- |
|  |  | Baseline | 3 month | 6 month | 12 month |  | Baseline | 3 month | 6 month | 12 month |
|  | | |  |  |  |  |  |  |  |  |
| **Severe and Diffuse Atherosclerosis** (n = 20) | | | | |  |  |  |  |  |  |
|  |  |  |  |  |  |  |  |  |  |  |
| Mean |  | 477 | 535 | 565 | 618 |  | 8.0 | 9.5 | 9.7 | 10.3 |
| ± SD |  | 61 | 61 | 63 | 90 |  | 1.7 | 1.1 | 0.8 | 1.4 |
| p* |  |  | <0.001 | <0.001 | <0.001 |  |  | 0.003 | <0.001 | <0.001 |
|  |  |  |  |  |  |  |  |  |  |  |
| **Moderate Atherosclerosis** ( n = 17) | | | |  |  |  |  |  |  |  |
|  |  |  |  |  |  |  |  |  |  |  |
| Mean |  | 485 | 539 | 547 | 641 |  | 7.3 | 8.3 | 8.6 | 9.2 |
| ± SD |  | 57 | 65 | 71 | 58 |  | 2.0 | 1.6 | 1.4 | 0.9 |
| p* |  |  | <0.001 | <0.001 | <0.001 |  |  | 0.003 | 0.005 | 0.003 |
|  |  |  |  |  |  |  |  |  |  |  |
| **Control (n=5)** | | | |  |  |  |  |  |  |  |
|  |  |  |  |  |  |  |  |  |  |  |
| Mean |  | 447 | 467 | 496 | 498 |  | 8.4 | 9.0 | 9.4 | 9.9 |
| ± SD |  | 22 | 32 | 53 | 26 |  | 1.4 | 1.0 | 0.6 | 0.2 |
| p* |  |  | 0.108 | 0.081 | 0.001 |  |  | 0.533 | 0.120 | 0.070 |
|  |  |  |  |  |  |  |  |  |  |  |
|  |  |  |  |  |  |  |  |  |  |  |
| p^†^ |  |  |  |  |  |  |  |  |  |  |
| Severe vs Moderate |  | 0.686 | 0.828 | 0.416 | 0.401 |  | 0.265 | 0.016 | 0.011 | 0.012 |
| Severe vs Control |  | 0.398 | 0.012 | 0.020 | <0.001 |  | 0.480 | 0.228 | 0.396 | 0.331 |
| Moderate vs Control |  | 0.159 | 0.014 | 0.159 | <0.001 |  | 0.273 | 0.497 | 0.161 | 0.010 |
|  |  |  |  |  |  |  |  |  |  |  |

*Wilcoxon signed rank statistic for change from baseline within group

^†^Wilcoxon rank sum statistic for differences between groups.

**Table D. Arterial Blood Pressure (mm Hg)**

|  |  | **Systolic** | | | |  | **Diastolic** | | | |
| --- | --- | --- | --- | --- | --- | --- | --- | --- | --- | --- |
|  |  | Baseline | 3 month | 6 month | 12 month |  | Baseline | 3 month | 6 month | 12 month |
|  | | |  |  |  |  |  |  |  |  |
| **Severe and Diffuse Atherosclerosis** (n = 20) | | | | |  |  |  |  |  |  |
|  |  |  |  |  |  |  |  |  |  |  |
| Mean |  | 153 | 152 | 160 | 160 |  | 100 | 102 | 105 | 108 |
| ± SD |  | 24 | 23 | 23 | 18 |  | 18 | 17 | 20 | 22 |
| p* |  |  | 0.937 | 0.230 | 0.218 |  |  | 0.728 | 0.380 | 0.173 |
|  |  |  |  |  |  |  |  |  |  |  |
| **Moderate Atherosclerosis** ( n = 17) | | | |  |  |  |  |  |  |  |
|  |  |  |  |  |  |  |  |  |  |  |
| Mean |  | 135 | 147 | 148 | 145 |  | 85 | 98 | 94 | 97 |
| ± SD |  | 21 | 18 | 22 | 20 |  | 15 | 20 | 18 | 19 |
| p* |  |  | 0.084 | 0.225 | 0.377 |  |  | 0.030 | 0.295 | 0.131 |
|  |  |  |  |  |  |  |  |  |  |  |
| **Control (n=5)** | | | |  |  |  |  |  |  |  |
|  |  |  |  |  |  |  |  |  |  |  |
| Mean |  | 140 | 151 | 142 | 151 |  | 91 | 102 | 93 | 102 |
| ± SD |  | 8 | 19 | 11 | 24 |  | 8 | 26 | 13 | 14 |
| p* |  |  | 0.180 | 0.806 | 0.203 |  |  | 0.322 | 0.707 | 0.025 |
|  |  |  |  |  |  |  |  |  |  |  |
|  |  |  |  |  |  |  |  |  |  |  |
| p^†^ |  |  |  |  |  |  |  |  |  |  |
| Severe vs Moderate |  | 0.039 | 0.468 | 0.140 | 0.038 |  | 0.020 | 0.594 | 0.086 | 0.132 |
| Severe vs Control |  | 0.296 | 0.935 | 0.075 | 0.538 |  | 0.454 | 0.836 | 0.208 | 0.856 |
| Moderate vs Control |  | 0.226 | 0.611 | 0.297 | 0.500 |  | 0.226 | 0.866 | 0.933 | 0.823 |
|  |  |  |  |  |  |  |  |  |  |  |

*Wilcoxon signed rank statistic for change from baseline within group

^†^Wilcoxon rank sum statistic for differences between groups.

**Table E. Fasting Aldosterone Levels (pg/ml)**

|  |  | Baseline | 3 month | 6 month | 12 month |
| --- | --- | --- | --- | --- | --- |
|  | | |  |  |  |
| **Severe and Diffuse Atherosclerosis** (n = 20) | | | |  |  |
|  |  |  |  |  |  |
| Mean |  | 163.3 | 182.3 | 183.6 | 203.9 |
| ± SD |  | 89.2 | 77.8 | 59.2 | 73.2 |
| p* |  |  | 0.056 | 0.042 | 0.037 |
|  |  |  |  |  |  |
| **Moderate Atherosclerosis** ( n = 17) | | | |  |  |
|  |  |  |  |  |  |
| Mean |  | 167.1 | 155.2 | 164.7 | 178.5 |
| ± SD |  | 101.1 | 87.8 | 85.2 | 80.3 |
| p* |  |  | 0.331 | 0.678 | 0.153 |
|  |  |  |  |  |  |
| **Control (n=5)** | | | |  |  |
|  |  |  |  |  |  |
| Mean |  | 158.8 | 167.0 | 170.9 | 153.8 |
| ± SD |  | 69.5 | 77.7 | 70.2 | 74.0 |
| p* |  |  | 0.773 | 0.487 | 0.650 |
|  |  |  |  |  |  |
|  |  |  |  |  |  |
| p^†^ |  |  |  |  |  |
| Severe vs Moderate |  | 0.922 | 0.180 | 0.208 | 0.288 |
| Severe vs Control |  | 0.869 | 0.530 | 0.668 | 0.265 |
| Moderate vs Control |  | 1.000 | 0.704 | 0.880 | 0.602 |
|  |  |  |  |  |  |

*Wilcoxon signed rank statistic for change from baseline within group

^†^Wilcoxon rank sum statistic for differences between groups.

**Table F. Fasting, 1 and 2 Hour Post Prandial Glucose levels (mg/dl) in Conscious Pigs**

|  |  | **Baseline** | | |  | **3 months** | | |  | **6 months** | | |  | **12 months** | | |
| --- | --- | --- | --- | --- | --- | --- | --- | --- | --- | --- | --- | --- | --- | --- | --- | --- |
|  |  | Pre | 1hr | 2hr |  | Pre | 1hr | 2hr |  | Pre | 1hr | 2hr |  | Pre | 1hr | 2hr |
|  |  |  |  |  |  |  |  |  |  |  |  |  |  |  |  |  |
| **Severe and Diffuse Atherosclerosis** (n = 13) | | | | | | | |  |  |  |  |  |  |  |  |  |
|  |  |  |  |  |  |  |  |  |  |  |  |  |  |  |  |  |
| Mean |  | 65 | 72 | 69 |  | 75 | 72 | 75 |  | 74 | 76 | 76 |  | 79 | 79 | 80 |
| ± SD |  | 9 | 9 | 9 |  | 7 | 8 | 12 |  | 5 | 8 | 10 |  | 12 | 10 | 17 |
| p* |  |  |  |  |  | 0.018 | 0.945 | 0.110 |  | 0.005 | 0.142 | 0.052 |  | 0.022 | 0.133 | 0.043 |
|  |  |  |  |  |  |  |  |  |  |  |  |  |  |  |  |  |
| **Moderate Atherosclerosis** (n = 6) | | | | |  |  |  |  |  |  |  |  |  |  |  |  |
|  |  |  |  |  |  |  |  |  |  |  |  |  |  |  |  |  |
| Mean |  | 64 | 71 | 67 |  | 69 | 68 | 74 |  | 73 | 74 | 82 |  | 73 | 76 | 77 |
| ± SD |  | 10 | 11 | 12 |  | 4 | 9 | 6 |  | 4 | 6 | 7 |  | 7 | 11 | 8 |
| p* |  |  |  |  |  | 0.144 | 0.403 | 0.193 |  | 0.106 | 0.431 | 0.071 |  | 0.156 | 0.444 | 0.173 |
|  |  |  |  |  |  |  |  |  |  |  |  |  |  |  |  |  |
| **Control** (n = 5) | | | | |  |  |  |  |  |  |  |  |  |  |  |  |
|  |  |  |  |  |  |  |  |  |  |  |  |  |  |  |  |  |
| Mean |  | 76 | 81 | 72 |  | 71 | 79 | 63 |  | 69 | 75 | 64 |  | 72 | 81 | 78 |
| ± SD |  | 5 | 11 | 10 |  | 6 | 7 | 11 |  | 6 | 10 | 13 |  | 14 | 20 | 13 |
| p* |  |  |  |  |  | 0.267 | 0.380 | 0.038 |  | 0.118 | 0.087 | 0.195 |  | 0.549 | 0.968 | 0.505 |
|  |  |  |  |  |  |  |  |  |  |  |  |  |  |  |  |  |
| p^†^ |  |  |  |  |  |  |  |  |  |  |  |  |  |  |  |  |
| Sev vs Mod |  | 0.985 | 0.881 | 0.914 |  | 0.101 | 0.623 | 0.652 |  | 0.986 | 0.619 | 0.111 |  | 0.728 | 0.664 | 0.909 |
| Sev vs Cont |  | 0.009 | 0.134 | 0.515 |  | 0.346 | 0.111 | 0.112 |  | 0.215 | 0.831 | 0.099 |  | 0.315 | 0.861 | 0.817 |
| Mod vs Cont |  | 0.017 | 0.268 | 0.831 |  | 0.411 | 0.162 | 0.126 |  | 0.190 | 0.887 | 0.017 |  | 0.823 | 0.890 | 0.968 |
|  |  |  |  |  |  |  |  |  |  |  |  |  |  |  |  |  |

*Wilcoxon signed rank statistic for change from baseline within group

^†^Wilcoxon rank sum statistic for differences between groups.

**Table G. Fasting, 1 and 2 Hour Post Prandial Insulin levels Insulin (μU/ml) in Conscious Pigs**

|  |  | **Baseline** | | |  | **3 months** | | |  | **6 months** | | |  | **12 months** | | |
| --- | --- | --- | --- | --- | --- | --- | --- | --- | --- | --- | --- | --- | --- | --- | --- | --- |
|  |  | Pre | 1hr | 2hr |  | Pre | 1hr | 2hr |  | Pre | 1hr | 2hr |  | Pre | 1hr | 2hr |
|  |  |  |  |  |  |  |  |  |  |  |  |  |  |  |  |  |
| **Severe and Diffuse Atherosclerosis** (n = 13) | | | | | | | |  |  |  |  |  |  |  |  |  |
|  |  |  |  |  |  |  |  |  |  |  |  |  |  |  |  |  |
| Mean |  | 10.7 | 32.5 | 28.7 |  | 17.5 | 38.6 | 32.4 |  | 20.4 | 32.6 | 31.1 |  | 36.0 | 32.2 | 39.3 |
| ± SD |  | 4.7 | 24.8 | 14.6 |  | 7.3 | 24.1 | 16.8 |  | 7.3 | 17.2 | 11.7 |  | 20.1 | 11.2 | 14.6 |
| p* |  |  |  |  |  | 0.006 | 0.507 | 0.496 |  | 0.001 | 0.998 | 0.567 |  | 0.001 | 0.771 | 0.082 |
| **Moderate Atherosclerosis** (n = 6) | | | | |  |  |  |  |  |  |  |  |  |  |  |  |
|  |  |  |  |  |  |  |  |  |  |  |  |  |  |  |  |  |
| Mean |  | 8.5 | 26.7 | 21.4 |  | 20.1 | 23.3 | 28.2 |  | 16.9 | 31.5 | 30.5 |  | 26.2 | 32.3 | 30.4 |
| ± SD |  | 4.7 | 17.4 | 10.9 |  | 7.5 | 7.6 | 7.9 |  | 10.5 | 20.0 | 212 |  | 7.2 | 18.2 | 12.0 |
| p* |  |  |  |  |  | 0.030 | 0.544 | 0.172 |  | 0.093 | 0.592 | 0.225 |  | 0.001 | 0.263 | 0.232 |
| **Control** (n = 5) | | | | |  |  |  |  |  |  |  |  |  |  |  |  |
|  |  |  |  |  |  |  |  |  |  |  |  |  |  |  |  |  |
| Mean |  | 15.5 | 38.8 | 35.9 |  | 17.1 | 64.7 | 40.0 |  | 22.3 | 62.6 | 34.2 |  | 39.7 | 72.0 | 58.6 |
| ± SD |  | 12.3 | 25.5 | 26.9 |  | 12.6 | 36.0 | 17.7 |  | 16.1 | 49.7 | 20.4 |  | 49.5 | 25.4 | 33.8 |
| p* |  |  |  |  |  | 0.766 | 0.114 | 0.772 |  | 0.306 | 0.251 | 0.909 |  | 0.258 | 0.076 | 0.395 |
|  |  |  |  |  |  |  |  |  |  |  |  |  |  |  |  |  |
| p^†^ |  |  |  |  |  |  |  |  |  |  |  |  |  |  |  |  |
| Sev vs Mod |  | 0.252 | 0.701 | 0.508 |  | 0.416 | 0.102 | 1.000 |  | 0.467 | 0.765 | 0.593 |  | 0.494 | 0.633 | 0.291 |
| Sev vs Cont |  | 1.000 | 0.443 | 0.758 |  | 0.503 | 0.167 | 0.336 |  | 0.924 | 0.289 | 1.000 |  | 0.279 | 0.006 | 0.383 |
| Mod vs Cont |  | 0.457 | 0.329 | 0.329 |  | 0.429 | 0.126 | 0.177 |  | 0.792 | 0.329 | 0.701 |  | 0.537 | 0.030 | 0.177 |

*Wilcoxon signed rank statistic for change from baseline within group

^†^Wilcoxon rank sum statistic for differences between groups.

**PONE-D-15-01878R1 NC3Rs ARRIVE Guidelines Checklist Final**


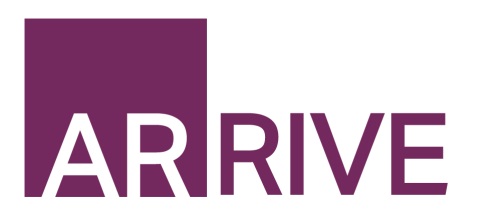


The ARRIVE Guidelines Checklist

Animal Research: Reporting In Vivo Experiments

Carol Kilkenny^1^, William J Browne^2^, Innes C Cuthill^3^, Michael Emerson^4^ and Douglas G Altman^5^

*^1^The National Centre for the Replacement, Refinement and Reduction of Animals in Research, London, UK, ^2^School of Veterinary Science, University of Bristol, Bristol, UK, ^3^School of Biological Sciences, University of Bristol, Bristol, UK, ^4^National Heart and Lung Institute, Imperial College London, UK, ^5^Centre for Statistics in Medicine, University of Oxford, Oxford, UK.*

|  | | ITEM | RECOMMENDATION | Section/ Paragraph |
| --- | --- | --- | --- | --- |
| 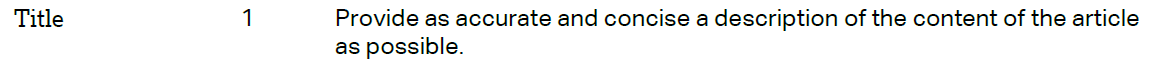 | | | Title, page 1 | |
| 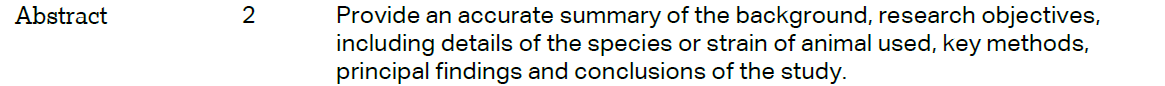 | | | Abstract, page 2 | |
| INTRODUCTION | | |  | |
| 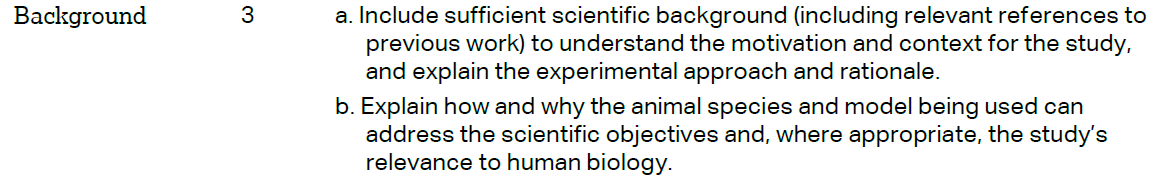 | | | a. Introduction page 3  b. Introduction pages 3 and 4, references 16 to 18. | |
| 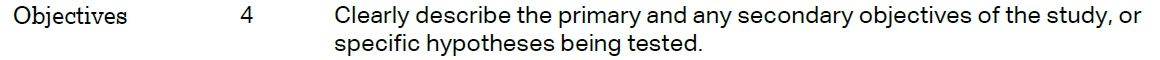 | | | Introduction, page 3, paragraph 2 and page 4, paragraph 1. | |
| METHODS | | |  | |
| 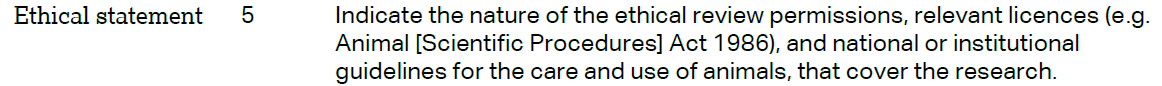 | | | Methods, page 5, paragraph 2, **Ethics Statement** | |
| 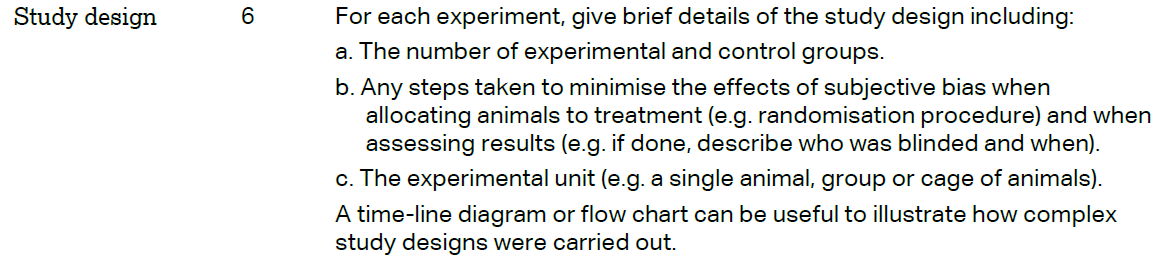 | | | a. Methods, page 4, paragraph 2, **Experimental Pigs** through page 5, paragraph 1 and Figures 1 and 2.  b and c. Methods, page 4, paragraph 2 through page 5 paragraph 1: “Forty-two pigs were entered into the year long study as they became available” and Figure 1 | |
| 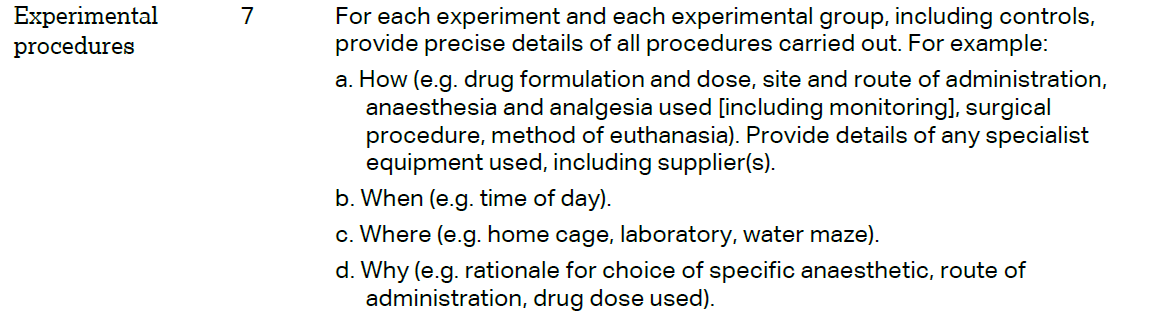 | | | a, b, c, d. Methods, page 5 paragraph 3 through page 6 paragraph 1. | |
| 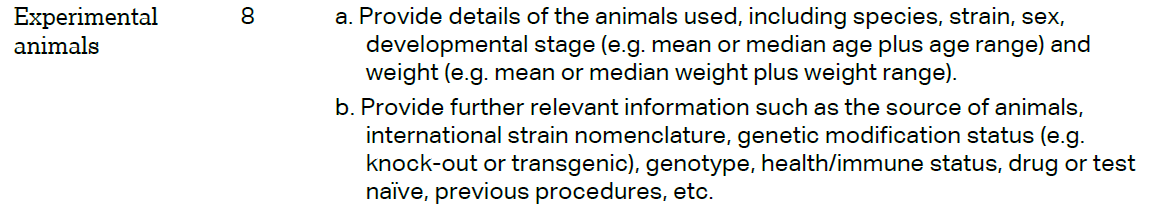 | | | a and b. Methods, **Experimental Pigs** page 4, paragraph 2, & page 5 paragraph 1, and S3 Table. | |

| 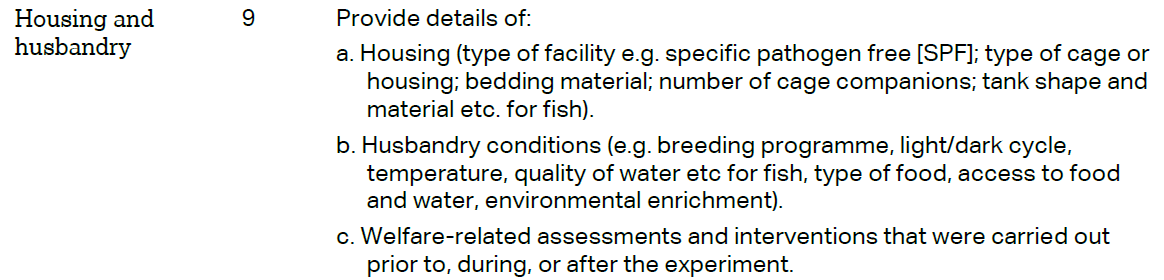 | a, b, c: Methods, **Experimental Pigs**,page 4, paragraph 2 and page 5 paragraph 1. | |
| --- | --- | --- |
| 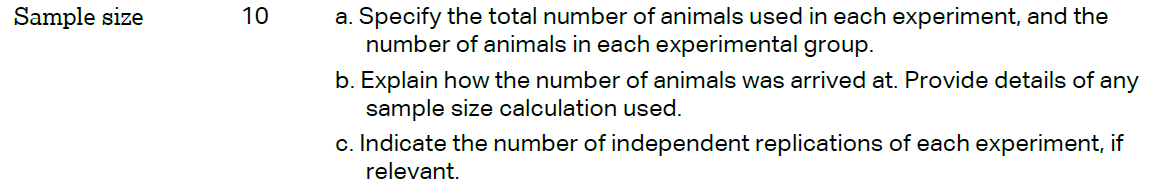 | a. Methods, **Experimental Pigs,** page 4, paragraph 2 and page 5 paragraph 1., & Figure1  b. Methods, page 4 paragraph 2, “Forty-two pigs were entered into the year long study as they became available.”  c. Not relevant | |
| 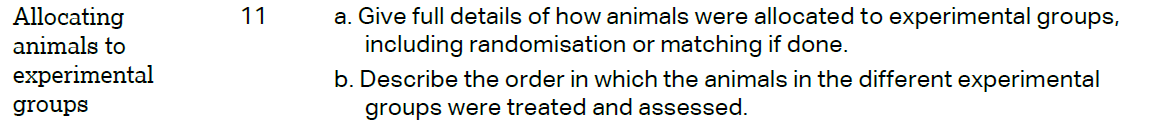 | a & b. Methods, page 4 paragraph 2, “Forty-two pigs were entered into the year long study as they became available” and Figure 1. | |
| 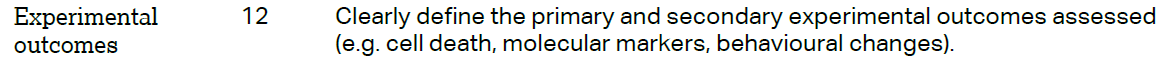 | Methods, page 10 **Biostatistical Analysis, “**The coronary intimal area as a percent of medial area was used as the primary measure of atherosclerotic severity.” | |
| 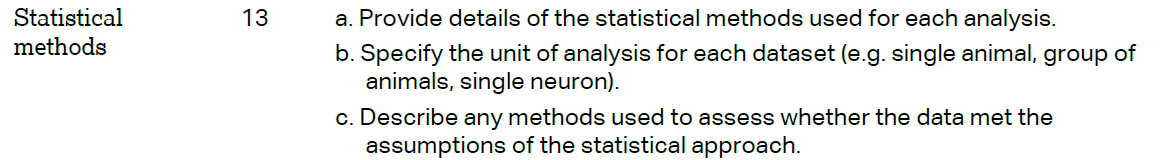 | a & b Methods, page 10 **Biostatistical Analysis**  c. Not applicable. | |
| RESULTS |  | |
| 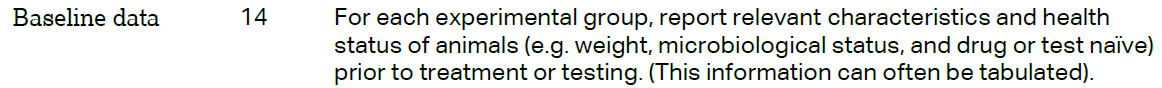 | Figure 1 and Tables 3 to 8 and S3 and S4 Tables | |
| 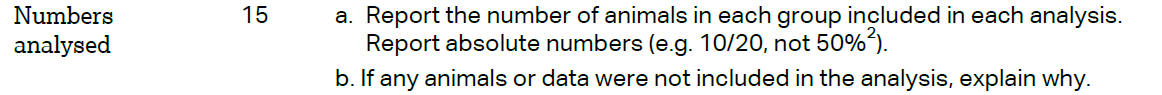 | a. Figures 1 and 2  b. No exclusions | |
| 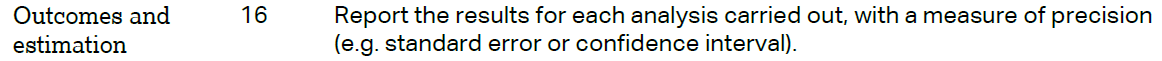 | Methods, page 8, paragraphs 1 and 2 and page 9 paragraph 1 | |
| 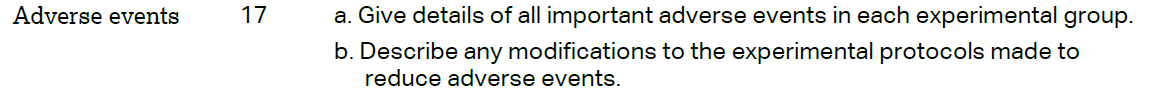 | a & b. last line page 17, **Adverse Events,** and first paragraph page 18. | |
| DISCUSSION |  | |
| 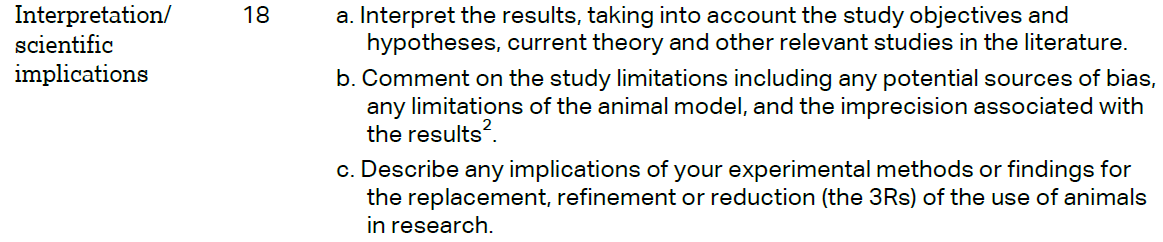 | a. **Discussion**, pages 18 – 27.  b. page 26, paragraph 2, **Study Limitations**  c. **Conclusion**, page 27, paragraph 2. | |
| 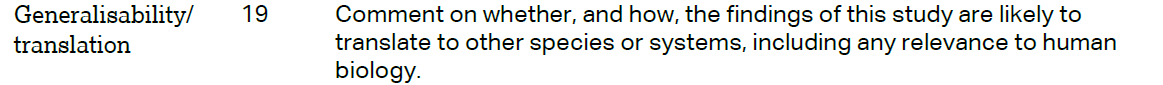 | **Conclusion**, page 27 | |
| 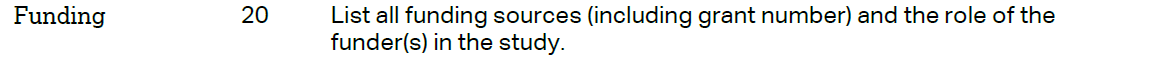 | | Title page lists Funding Support and and Role of Funders |


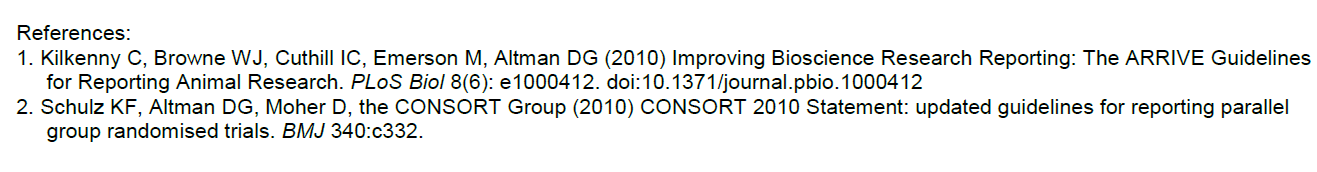

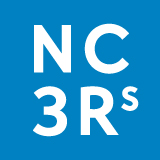

Supplement: S1 File — (DOCX) [file pone.0132302.s001.docx]
